# Exosomes as Emerging Therapeutic and Diagnostic Platforms: Biological Functions, Clinical Applications, and Translational Challenges

**DOI:** 10.3390/ijms27156979

**Published:** 2026-08-03

**Authors:** Chin-Yin Lin, Chih-Yang Lin, Woei-Cherng Shyu, Long-Bin Jeng, Syuan-Ling Lin

**Affiliations:** 1 Translational Medicine Research Center, China Medical University Hospital, Taichung 404, Taiwan; 2 Translational Medicine Center, Shin-Kong Wu Ho-Su Memorial Hospital, Taipei 111, Taiwan; 3 Graduate Institute of Biomedical Sciences, China Medical University, Taichung 411, Taiwan; 4 Cell Therapy Center, China Medical University Hospital, Taichung 404, Taiwan; 5 Organ Transplantation Center, China Medical University Hospital, Taichung 404, Taiwan

**Keywords:** extracellular vesicles, exosome biogenesis and cargo sorting, engineered exosomes, targeted drug delivery, translational hurdles, precision medicine

## Abstract

Exosomes are small membrane-bound extracellular vesicles of endosomal origin that play pivotal roles in intercellular communication by transferring proteins, lipids, metabolites, and nucleic acids. Increasing evidence indicates that these vesicles participate in diverse physiological and pathological processes, including immune regulation, tissue repair, tumor progression, neurodegeneration, and cardiovascular homeostasis. Their distinctive biological properties have consequently generated considerable interest in their development as diagnostic tools, therapeutic agents, and drug-delivery platforms. Recent progress in exosome biology and biogenesis, together with technological advances in vesicle isolation, characterization, engineering, and large-scale production, has accelerated their preclinical development and early clinical translation. Nevertheless, substantial challenges—including donor-cell heterogeneity, low production yields, insufficiently standardized protocols, and regulatory uncertainty—must be addressed before widespread clinical implementation can be achieved. This narrative review integrates current knowledge of exosome biology, diagnostic and therapeutic applications, and engineering strategies. It further examines major translational barriers and outlines future priorities for advancing exosome-based technologies toward precision medicine.

## 1. Introduction

**Figure 1:**
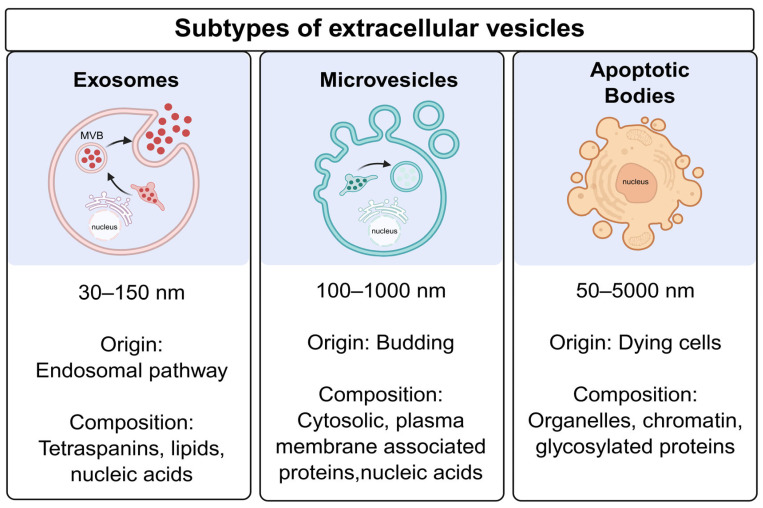
Classification, biogenesis, and characteristics of extracellular vesicle (EV) subtypes. EVs are heterogeneous, membrane-bound particles broadly categorized into three major populations based on their size, origin, and molecular composition. (**Left**) Exosomes (30–150 nm) originate from the endosomal pathway via the formation of multivesicular bodies (MVBs) and are enriched in tetraspanins, lipids, and nucleic acids. (**Middle**) Microvesicles (100–1000 nm) are generated through outward budding of the plasma membrane, carrying cytosolic components, plasma membrane-associated proteins, and nucleic acids. (**Right**) Apoptotic bodies represent the largest subtype (50–5000 nm), released from dying cells undergoing apoptosis; they typically contain cellular organelles, chromatin fragments, and glycosylated proteins. This schematic summarizes the defining physical and biological properties of these principal EV subpopulations.

Secreted by virtually all cell types, exosomes are membrane-bound extracellular vesicles (EVs) that serve as vital mediators of intercellular communication. Representing a distinct subtype of EVs derived from the endosomal pathway, exosomes (30–150 nm in diameter) function as active vehicles that transport a complex array of bioactive cargoes, including proteins, lipids, metabolites, and genomic components (mRNAs, miRNAs, lncRNAs, circRNAs, and DNA) [1,2] (Figure 1).

**Figure 2:**
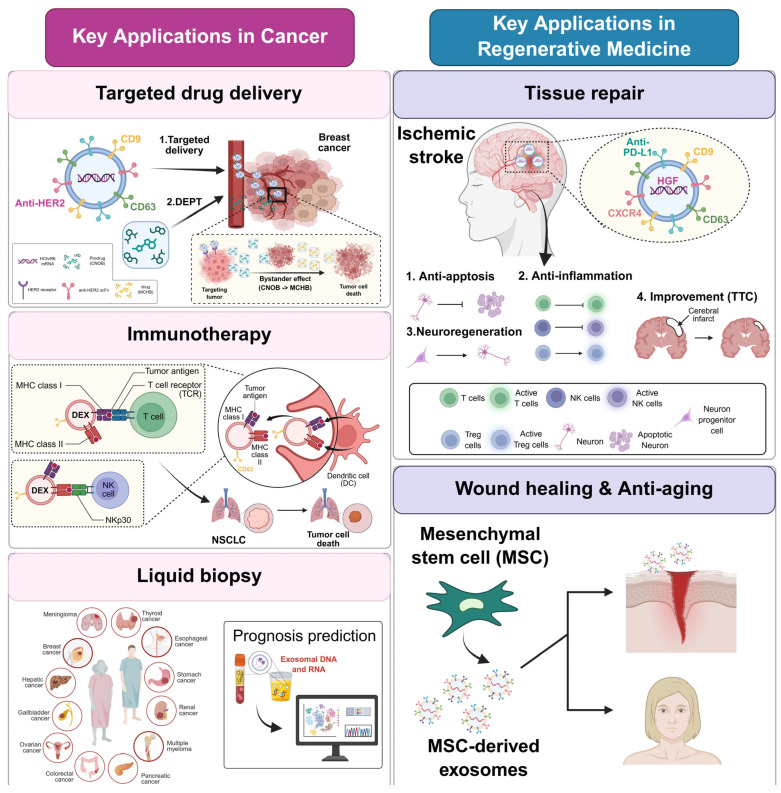
Representative diagnostic and therapeutic applications of exosomes in oncology and regenerative medicine. Exosomes have emerged as versatile nanoscale vesicles with broad biomedical potential owing to their biocompatibility, membrane-associated targeting properties, and capacity to carry nucleic acids, proteins, and therapeutic agents. In oncology, engineered exosomes can facilitate tumor-targeted drug delivery, thereby enhancing therapeutic efficacy while reducing off-target toxicity. Dendritic cell-derived exosomes (DEXs) carrying tumor antigens and major histocompatibility complex (MHC) molecules can prime T cells and activate natural killer (NK) cells, thereby promoting antitumor immune responses [7,8]. Exosomal DNA and RNA isolated from biological fluids may also serve as minimally invasive biomarkers for cancer detection, liquid biopsy, and prognostic assessment. In regenerative medicine, therapeutic exosomes may promote tissue repair through anti-apoptotic, anti-inflammatory, and neuroregenerative mechanisms. In ischemic stroke, engineered exosomes incorporating CXCR4, HGF, and anti-PD-L1-related components can enhance brain targeting, attenuate cerebral injury, and promote neurological recovery [9]. In addition, mesenchymal stem cell-derived exosomes show promising potential in wound healing, tissue regeneration, and anti-aging applications. This schematic summarizes representative diagnostic and therapeutic applications of exosomes in oncology and regenerative medicine.

The delivery of these macromolecules facilitates local and systemic cellular crosstalk essential for maintaining physiological homeostasis. Driven by their multifaceted roles, exosomes have attracted considerable research attention over the past decade. Increasing evidence demonstrates that exosomes participate in diverse physiological events, including immune surveillance, tissue regeneration, angiogenesis, neuronal communication, and organ development. Because their molecular cargo dynamically reflects the physiological and pathological states of the donor cells, exosomes can transmit cell-specific signals to recipient cells, thereby contributing to the regulation of cellular homeostasis. For instance, within the immunological niche, they modulate antigen presentation, T-cell activation, macrophage polarization, and cytokine cascades [3,4,5]. Furthermore, mesenchymal stem cell (MSC)-derived exosomes have demonstrated potent pro-survival, proliferative, angiogenic, and neuroplastic effects to drive tissue repair and extracellular matrix remodeling [6]. By delivering functional cargoes to recipient cells, exosomes mediate intercellular communication and influence immune regulation, tissue repair, and disease progression. These biological properties provide the foundation for their emerging diagnostic and therapeutic applications in oncology and regenerative medicine, as summarized in Figure 2.

### 1.1. Pathogenesis and Disease Progression

Beyond their homeostatic functions, exosomes are deeply implicated in the pathogenesis and exacerbation of numerous disorders. In oncology, tumor-derived exosomes actively fuel virtually every stage of malignancy, including proliferation, angiogenesis, immune evasion, epithelial–mesenchymal transition (EMT), metastasis, and chemoresistance [10,11]. By secreting specific molecular cargoes, malignant cells remodel the local tumor microenvironment and scaffold pre-metastatic niches that prime distant tissues for secondary colonization [12,13]. Within the central nervous system, these vesicles mediate the propagation of proteopathic aggregates, such as amyloid-β, tau, and α-synuclein, thereby perpetuating the progression of Alzheimer’s disease (AD) and Parkinson’s disease (PD) [14,15]. For example, neuron-derived exosomes carrying phosphorylated tau have been detected in the plasma of patients with AD and exhibit strong correlations with disease severity and cognitive decline, highlighting their potential as minimally invasive biomarkers for early diagnosis and disease monitoring. Likewise, exosomal α-synuclein released from neurons has been shown to promote the spread of pathological protein aggregates and neurotoxicity in PD, further supporting the critical role of exosome-mediated intercellular communication in neurodegeneration. Furthermore, dysregulated exosomal signaling underpins the etiology of autoimmune disorders [16], infectious diseases [17], metabolic syndromes [18], and chronic inflammatory conditions. For instance, in various inflammatory disease models, mesenchymal stem cell-derived exosomes have been shown to attenuate inflammatory responses by promoting macrophage polarization toward an anti-inflammatory M2-like phenotype [5,19,20], whereas tumor-derived exosomes frequently establish an immunosuppressive microenvironment that facilitates immune evasion and disease progression [19]. Collectively, these multifaceted biological functions underscore the dual roles of exosomes as both drivers of disease pathogenesis and promising therapeutic targets.

### 1.2. Diagnostic Applications

Moreover, their remarkable stability, protection of encapsulated biomolecules, and accessibility in diverse biological fluids have stimulated intensive investigation into their clinical applications, particularly as minimally invasive biomarkers for disease diagnosis, prognosis, and therapeutic monitoring. Because exosomes are abundantly shed into various biofluids—including blood, urine, saliva, cerebrospinal fluid, and synovial fluid—they can be readily harvested via liquid biopsies. Crucially, the encapsulated cargo preserves the molecular blueprints of the parental cells, offering real-time insights into disease initiation, progression, and treatment response. Supported by recent breakthroughs in high-throughput sequencing, proteomics, and metabolomics, the profiling of disease-specific exosomal signatures has revolutionized early detection, patient stratification, and the broader landscape of precision medicine.

### 1.3. Therapeutic Applications

Beyond diagnostics, exosomes represent promising therapeutic modalities and versatile drug delivery platforms. In contrast to synthetic counterparts, exosomes offer inherent biological merits, such as excellent biocompatibility, low immunogenicity, stability in biofluids, and the ability to breach tight biological barriers (e.g., the blood–brain barrier, BBB) [21,22]. These features make them ideal for trafficking functional cargoes, including nucleic acids, proteins, peptides, small molecules, and gene-editing components. Recent bioengineering innovations have fostered the development of next-generation exosomes with enhanced targeting, improved loading efficiency, and superior therapeutic efficacy. Harnessing surface conjugation, genetic manipulation, and advanced encapsulation techniques has expanded their applicability across diverse disease models. Concurrently, stem cell-derived exosomes have shown robust regenerative and immunomodulatory potency in preclinical models of tissue injury, ischemia, and inflammation, propelling these therapies toward clinical trials [23,24].

### 1.4. Translational Obstacles

However, formidable challenges still impede the clinical translation of exosome-based technologies. A major translational challenge is vesicle heterogeneity, which is influenced by donor-cell source, culture conditions, and isolation and purification workflows. This variability complicates batch-to-batch consistency, quality control, regulatory standardization, and the reliable assessment of therapeutic efficacy and safety [25,26]. Moreover, suboptimal production yields restrict large-scale manufacturing. Developing reproducible cargo-loading strategies is also a persistent challenge, as existing techniques often lead to low encapsulation efficiency or compromised vesicle structure. Crucially, the exact biodistribution, pharmacokinetics, long-term safety, and off-target risks of exosome formulations remain incomplete. Driven by the rapid pace of this field, a comprehensive evaluation of current milestones and persistent barriers is highly timely. Specifically, this narrative review aims to address a central research question: How can native and bioengineered exosomes be harnessed as reliable diagnostic and therapeutic platforms, and what critical hurdles must be overcome to realize their clinical translation in precision medicine? To answer this, this review provides a structured overview of exosome biogenesis and characterization, critically evaluates advanced cargo and surface engineering strategies, and dissects current translational bottlenecks—including large-scale manufacturing, yield optimization, and regulatory standardization—to illuminate future perspectives for exosome-based precision pharmacotherapy.

## 2. Isolation, Purity and Quality Assessment of Exosomes

### 2.1. Non-EV Contaminants and Limitations in Exosome Isolation

Exosome preparations isolated from cell culture-conditioned media and biological fluids often contain non-vesicular components, including lipoproteins, soluble proteins, protein aggregates, and residual cellular debris. These contaminants may overlap with EVs in size, density, sedimentation behavior, or other physicochemical properties and can therefore be co-isolated during ultracentrifugation, polymer-based precipitation, or size-based enrichment. Their presence can lead to overestimations of particle concentrations and significant distortions in proteomic, lipidomic, and nucleic acid profiles [27]. Furthermore, it may trigger EV-independent biological effects, including metabolic, inflammatory, or immune responses. This issue is particularly relevant to plasma- and serum-derived preparations, in which abundant lipoproteins and soluble proteins may interfere with EV isolation and downstream analyses [28]. Accordingly, sample purity should be evaluated using both EV-associated proteins and representative contaminant markers, such as APOA1, APOB, and APOE for lipoproteins; albumin for soluble plasma proteins; and calnexin or GM130 for cellular components [29]. Because no single isolation technique can completely separate EVs from all non-EV structures, complementary purification approaches are generally required. Differential centrifugation and filtration can reduce cells and cellular debris, whereas size-exclusion chromatography and density-gradient centrifugation can improve the removal of soluble proteins, protein aggregates, and lipoproteins. Functional studies should additionally include mock-isolation controls, matched non-EV fractions, or EV-depleted controls to avoid erroneously attributing contaminant-mediated responses to exosomes. Common non-EV contaminants, their potential interference, representative detection markers, and mitigation strategies are summarized in Table 1.

### 2.2. Characterization and Quality Assessment of Exosomes

Biophysical and morphological characterization is essential for evaluating the size distribution, particle concentration, structural integrity, and heterogeneity of exosome preparations. Nanoparticle tracking analysis and tunable resistive pulse sensing are commonly employed to determine particle size and concentration, whereas dynamic light scattering provides information regarding the average hydrodynamic diameter and polydispersity of vesicle populations. Transmission electron microscopy enables direct visualization of vesicle morphology, while cryogenic electron microscopy preserves the native membrane structure and reduces preparation-associated artifacts. Atomic force microscopy can additionally provide information regarding vesicle surface topography and mechanical properties. Nevertheless, particle-based methods cannot independently distinguish exosomes from other extracellular vesicle subtypes, lipoproteins, or protein aggregates with overlapping size ranges. Therefore, biophysical and morphological analyses should be interpreted in combination with complementary molecular assessments [1,30,31]. Biochemical and molecular characterization provides critical information regarding vesicle identity, cellular origin, and sample purity. Western blotting, enzyme-linked immunosorbent assays, bead-based flow cytometry, and single-particle immunoassays are widely used to detect EV-associated proteins. Commonly assessed markers include the tetraspanins CD9, CD63, and CD81, together with proteins associated with endosomal biogenesis, such as TSG101, ALIX, and syntenin-1. In parallel, the presence of cellular or non-vesicular contaminants should be evaluated using markers such as calnexin, GM130, albumin, apolipoprotein A1, and apolipoprotein B. Proteomic comparisons have demonstrated that conventionally used EV markers may exhibit heterogeneous distributions among distinct EV populations and donor cell types. Consequently, the presence or absence of a single marker is insufficient to establish exosome identity or purity, and multiple markers representing different protein categories should be evaluated [32,33].

Exosomal cargo analysis encompasses the characterization of nucleic acids, proteins, lipids, and metabolites. Quantitative PCR, digital PCR, and RNA sequencing are commonly applied to profile miRNAs, mRNAs, and other RNA species, whereas mass spectrometry-based proteomics, lipidomics, and metabolomics enable broader molecular characterization. Appropriate controls, including nuclease or protease treatment with and without membrane disruption, may help distinguish intravesicular cargo from molecules adsorbed onto the vesicle surface or present as co-isolated contaminants. Accordingly, reliable quality assessment requires orthogonal analytical approaches that collectively evaluate particle properties, morphology, molecular markers, purity, and cargo composition. Consistent with the MISEV2023 recommendations, the selection of characterization methods should be tailored to the biological source, isolation procedure, and intended diagnostic or therapeutic application [34,35].

## 3. Dual Roles of Exosomes in Homeostasis and Disease

Exosomes exert context-dependent biological effects determined by their cellular origin, cargo composition, and the physiological state of recipient cells. Under normal conditions, immune cell-derived exosomes contribute to antigen presentation and immune cell activation, whereas mesenchymal stem cell-derived exosomes support tissue repair by promoting angiogenesis, suppressing apoptosis, and modulating inflammatory responses [5,23,36,37]. These functions illustrate the physiological roles of exosomes in maintaining tissue homeostasis and coordinating intercellular communication. Conversely, disease-associated exosomes can propagate pathological signals and remodel recipient tissues. Tumor-derived exosomes promote angiogenesis, immune evasion, epithelial–mesenchymal transition, metastatic niche formation, and therapeutic resistance [38,39]. In neurodegenerative disorders, exosomes facilitate the intercellular transmission of pathological proteins, including amyloid-β, tau, and α-synuclein [14,15]. Dysregulated exosomal signaling has also been implicated in autoimmune, metabolic, and chronic inflammatory disorders [16,18]. Collectively, these findings underscore the dual roles of exosomes as physiological mediators and pathological effectors.

## 4. Exosomes as Next-Generation Diagnostic Biomarkers

Because exosomal cargo can reflect the molecular characteristics and physiological state of their cells of origin, exosomes have attracted considerable interest as minimally invasive biomarkers [40,41]. Present in almost all biological fluids—including blood, urine, saliva, cerebrospinal fluid, breast milk, and synovial fluid—exosomes are readily obtainable via liquid biopsy. Furthermore, their lipid bilayer membrane protects encapsulated cargo from circulating nucleases and proteases, ensuring the structural integrity of critical disease indicators. Recent advances in multi-omics technologies have enabled deep profiling of exosomal proteins, RNAs, lipids, and metabolites. In particular, exosomal miRNAs represent attractive diagnostic tools due to their exceptional stability and signature expression patterns in disease states. Comprehensive research has validated exosomal miRNA panels for cancer detection, staging, and monitoring treatment responses [42]. Concurrently, exosomal proteins like PD-L1, EGFR, HER2, and glypican-1 have shown robust diagnostic accuracy in various malignancies [43]. The clinical utility of exosome diagnostics extends across multiple medical specialties. In neurodegeneration, exosomal tau and amyloid-related proteins serve as promising candidates for the preclinical detection of Alzheimer’s disease. In cardiovascular disorders, circulating exosomal miRNA profiles strongly correlate with myocardial injury, heart failure, and atherosclerosis. Similarly, quantifying exosomal inflammatory cytokines allows for the real-time tracking of autoimmune disease flares. Integrating liquid biopsy methods with next-generation sequencing, advanced proteomics, and artificial intelligence-driven data analytics has accelerated the development of smart diagnostic algorithms. Although several exosomal nucleic acid and protein biomarkers have demonstrated promising diagnostic performance in human samples, substantial heterogeneity in specimen type, exosome isolation and enrichment procedures, analytical platforms, and cohort design currently limits cross-study comparability and clinical validation. Accordingly, standardized isolation protocols, harmonized analytical workflows, improved cross-platform sensitivity and assay reproducibility, and prospective multicenter validation will be essential before exosome-based diagnostic assays can be adopted in routine clinical practice.

## 5. Exosomes as Therapeutic Agents and Drug Delivery Platforms

The intrinsic pharmacological merits of naive exosomes have positioned them at the forefront of biomimetic pharmacotherapy and cell-free regenerative medicine [44]. Unlike conventional synthetic nanoparticles, these cell-derived exosomes exhibit evolutionarily refined biological features, including superior biocompatibility, minimal immunogenicity, prolonged circulation kinetics, and the exceptional capacity to breach restrictive physiological barriers. These unique attribute profiles have galvanized intensive exploration into exosome-based therapeutics across a multifaceted spectrum of pathologies. A premier paradigm within regenerative medicine focuses on MSC-derived exosomes. Accumulating evidence underscores that these exosomes naturally encapsulate a therapeutic arsenal of growth factors, cytokines, and microRNAs, as well as regulatory non-coding RNAs capable of orchestrating complex tissue repair cascades [45]. Robust therapeutic efficacy of these naive MSC exosomes has been widely demonstrated across various preclinical models of organ injury and degenerative conditions. Crucially, exosome-based intervention serves as a compelling cell-free alternative that successfully circumvents the critical safety and translational bottlenecks inherent to traditional stem cell transplantation, such as low post-transplantation engraftment rates, potential tumorigenicity (malignant transformation), and host immune rejection.

In the oncology field, exosomes have captured significant attention as both direct anti-tumor agents and highly adaptable vehicles for targeted drug delivery. Because of their innate immuno-evasive properties—which prevent rapid clearance by the mononuclear phagocyte system—these natural exosomes serve as an ideal pharmacological platform to transport cytostatic drugs or regulatory nucleic acids directly into the malignant niche. By leveraging selective targeted formulations, exosome-mediated delivery drastically amplifies therapeutic potency within tumor tissues while mitigating the severe off-target systemic toxicities commonly associated with conventional chemotherapy [46,47,48].

Concurrently, exosome-mediated immunotherapy has emerged as a rapidly developing therapeutic field. Dendritic cell-derived exosomes (DEXs) have been investigated as cell-free cancer vaccines because they can present tumor-associated antigens and major histocompatibility complex molecules to stimulate T-cell- and natural killer cell-mediated antitumor responses. In parallel, engineered exosomal platforms can be designed to display immunomodulatory ligands or deliver therapeutic cargoes, thereby enhancing antitumor immunity or attenuating dysregulated inflammatory responses in autoimmune and inflammatory diseases [49]. Representative clinical trials and pivotal preclinical studies of exosome-based therapeutic platforms are summarized in Table 2. Current clinical evidence is derived mainly from early-phase studies of DEX-based immunotherapy, whereas most cargo- and surface-engineered exosome platforms remain at the preclinical stage. Although these studies demonstrate promising safety, targeting, immunomodulatory, and therapeutic effects, the strength and translational relevance of the available evidence vary according to study design. Therefore, findings from in vitro and animal studies should not be interpreted as equivalent to demonstrated clinical efficacy, and further well-designed clinical trials are required to establish pharmacokinetics, therapeutic efficacy, long-term safety, and an appropriate therapeutic window.

## 6. Engineered Exosomes and Emerging Technologies

While naive exosomes possess intrinsic evolutionary benefits—such as superior biocompatibility, low immunogenicity, and innate intercellular communication networks—their clinical translation is significantly bottlenecked by native architectural constraints. These limitations include heterogeneous cargo encapsulation, poor tissue tropism, limited therapeutic potency, and suboptimal manufacturing yields. To address these hurdles, extensive research has shifted toward bioengineering strategies aimed at developing next-generation exosomal platforms with engineered biological profiles and augmented delivery kinetics. Recent advances have enabled the precise customization of these exosomes through optimized cargo sorting, surface functionalization, parental cell reprogramming, and integration with cutting-edge nanotechnologies.

### 6.1. Cargo Engineering Strategies

A primary focus in enhancing therapeutic efficacy involves the strategic modification of exosomal payloads [59,60]. This can be executed either by engineering donor cells prior to vesicle shedding (pre-loading) or by artificially introducing therapeutic agents into isolated vesicles (post-loading). Pre-loading methodologies typically utilize genetic modification to drive the cellular overexpression of therapeutic proteins, peptides, or critical RNA payloads, ranging from coding mRNAs to regulatory non-coding species (miRNAs, lncRNAs, and circRNAs). During endosomal trafficking, these molecules are selectively sorted into the invaginating intraluminal vesicles for subsequent delivery [9]. This genetic hijacking of cellular factories has shown robust therapeutic efficacy in regenerative medicine and oncology [53]; for example, engineering vesicles to enrich specified miRNAs (e.g., miR-124 [54,61], miR-21 [57], or miR-146a [55]) has successfully ameliorated neurological deficits, myocardial ischemia, and systemic inflammatory cascades in preclinical models. Conversely, post-loading strategies capitalize on artificial drug encapsulation into exosomal membranes [62]. Common modalities encompass physical disruption techniques such as electroporation, sonication, extrusion, and freeze–thaw cycles, which are frequently complemented by chemical permeabilization or saponin-assisted loading, dialysis, and passive incubation. Additionally, microfluidics-based loading systems have recently gained prominence as a highly controlled platform to facilitate payload incorporation. Although electroporation remains a staple for nucleic acid loading, persistent concerns regarding cargo aggregation and irreversible membrane damage frequently compromise vesicle integrity [62,63,64]. The principal advantages and limitations of these strategies are summarized in Table 3.

### 6.2. Surface Engineering for Enhanced Tissue Tropism

Although certain naive exosomes exhibit limited, cell-derived organotropism—largely mediated by specific surface adhesion molecules such as integrins—their natural homing efficiency is profoundly inadequate for systemic precision therapies. Upon systemic administration, the vast majority of un-engineered vesicles undergo rapid sequestration by the mononuclear phagocyte system (MPS), accumulating non-specifically in the liver and spleen. This rapid clearance, combined with the lack of robust cell-type specificity, leaves naive vesicles ineffective for targeting extrahepatic malignancies or localized tissue lesions. Consequently, Surface engineering has become an indispensable cornerstone for circumventing these clearance mechanisms and endowing exosomes with active, ligand-mediated homing capabilities required for true cellular precision medicine. To achieve programmatic active targeting, genetic engineering of donor cells allows for the designed display of homing ligands directly on the exosomal membrane. This is routinely executed by constructing chimeric genes that fuse targeting peptides to the accessible ectodomains of highly abundant exosomal scaffolding proteins, such as Lamp2b, CD63, CD9, or CD81 [65]. Upon biogenesis, these tailormade vesicles possess the molecular architecture required to selectively engage disease-associated receptors, thereby drastically optimizing tissue-specific accumulation. A classic example of genetic surface engineering is the RVG-functionalized exosome, in which donor cells are engineered to express an RVG–Lamp2b fusion protein on the exosomal membrane. These vesicles facilitate targeted delivery of therapeutic nucleic acids to the brain following systemic administration [22], with subsequent studies indicating that RVG promotes BBB penetration through receptor-associated endocytosis and transcytotic trafficking [66]. Alternatively, bio-orthogonal chemical modification provides a highly versatile, non-genetic avenue for post-isolation vesicle functionalization. Advanced techniques—comprising copper-free click chemistry, covalent bioconjugation, hydrophobic lipid membrane insertion, and aptamer anchoring—are widely deployed to decorate the vesicular envelope with monoclonal antibodies, homing peptides, or molecular imaging contrast agents [67,68,69]. These chemoselective modifications not only sharpen active-targeting specificity but also pave the way for sophisticated theranostic applications by seamlessly coupling high-resolution molecular imaging with site-specific payload delivery. More recently, antibody-functionalized exosomes (conceptually designated as nano-bites) displaying binders that target tumor-associated antigens or immune checkpoint ligands have garnered intense interest [56,58,70]. These platforms have demonstrated exceptional accumulation within the immunosuppressive tumor microenvironment, effectively modernizing drug delivery by eliciting robust, synergistically enhanced antitumor responses in rigorous preclinical evaluations.

### 6.3. Parental Cell Reprogramming

Because the biological signature and therapeutic archetype of an exosome are largely dictated by its cell of origin, engineering the parental cells represents a radical upstream strategy for customizing vesicle functionality at the source. Rather than manipulating harvested vesicles ex vivo, genetically re-engineered stem cells, immune cells, and malignant lines can be established as specialized cellular factories or programmable bioreactors to mass-produce functionalized vesicles. Among available options, MSCs remain the premier producer cell line owing to their intrinsic immunomodulatory properties, low immunogenicity, and exceptionally high baseline secretory capacity [71,72]. Furthermore, the integration of CRISPR/Cas9 gene-editing toolkits has revolutionized parental cell engineering, allowing researchers to precisely knock in or knock out specific genomic loci. This precise editing serves to optimize endogenous cargo packaging networks, amplify vesicle secretion rates, and alter membrane tropism before the vesicles are even shed. Simultaneously, synthetic biology has emerged as an innovative paradigm for designing programmable, intelligent exosome-producing cells. By embedding artificial genetic circuits within these parental hosts, researchers can achieve dynamic, stimuli-responsive control over exosome biogenesis and custom payload loading. This synthetic framework empowers the production cells to precisely turn vesicle secretion on or off, or to selectively alter cargo composition, in autonomous response to specific pathological microenvironmental cues or exogenous chemical triggers.

### 6.4. Emerging Computational and Manufacturing Technologies

The rapid evolution of exosome engineering is intrinsically tied to a convergence with other revolutionary technologies. Artificial intelligence (AI) and deep machine learning are increasingly harnessed to mine complex exosomal multi-omics datasets, predict optimal multi-cargo synergy, and optimize the topological design of synthetic homing ligands, thereby accelerating the pipeline of personalized vesicle therapeutics. Concurrently, microfluidic technologies have emerged as promising tools for EV isolation and analysis in specialized academic and translational research laboratories. Representative platforms, including EXODUS, ExoTIC, and ExoChip, enable rapid processing of small sample volumes and can integrate EV enrichment with downstream characterization [73,74,75,76]. However, these technologies are not yet routinely adopted across most general academic laboratories, where ultracentrifugation and size-exclusion chromatography remain more commonly used. Broader implementation is currently limited by platform heterogeneity, requirements for specialized fabrication or instrumentation, cost, and the need for improved interlaboratory standardization and large-scale validation [77,78]. Finally, the development of exosome-encapsulated gene-editing systems represents a promising frontier. By delivering CRISPR/Cas9 ribonucleoprotein complexes or mRNA therapeutics via engineered vesicles, researchers can achieve high-efficiency gene editing in target tissues with a significantly reduced risk of immunogenicity and off-target effects compared to conventional adenoviral or adeno-associated viral (AAV) vectors. Collectively, this multifaceted progress—encompassing cargo refinement, surface functionalization, parental cell reprogramming, biomimetic architecture, and computational intelligence—has broadened the therapeutic paradigm of exosome medicine, accelerating the transition of these nanovesicles into clinical translation.

### 6.5. Storage and Preservation of Exosomes

Storage and preservation conditions are critical determinants of exosome stability, reproducibility, and translational performance. When immediate analysis is not feasible, isolated small EVs may be stored at 4 °C or −20 °C for short-term preservation depending on the intended application, whereas −80 °C is generally more suitable for long-term storage [79,80]. Nevertheless, freezing and repeated freeze–thaw cycles may alter particle recovery, size distribution, membrane integrity, molecular cargo, and biological activity. Therefore, samples should preferably be aliquoted before freezing to minimize repeated freeze–thaw exposure [80,81]. The inclusion of stabilizing excipients can reduce storage-associated damage; for example, trehalose has been shown to suppress vesicle aggregation and improve the retention of biological activity during cryopreservation [81].

Lyophilization represents an alternative preservation strategy that may reduce dependence on continuous cold-chain transportation and facilitate the distribution of exosome-based products. However, lyophilization without suitable protective excipients may promote vesicle aggregation and compromise recovery after reconstitution. Formulations containing trehalose or sucrose can improve colloidal stability, cargo retention, and functional activity following freeze-drying [82]. Because preservation outcomes vary according to the EV source, isolation procedure, buffer composition, particle concentration, and cargo properties, storage protocols should be established and validated for each formulation. Post-storage quality assessment should include particle concentration and size, morphology, surface-marker expression, cargo integrity, cellular uptake, and an application-relevant potency assay to confirm retention of the intended biological function [80,83].

## 7. Conclusions

Exosomes represent promising biological platforms for minimally invasive diagnosis, targeted drug delivery, immunomodulation, and cell-free regenerative therapy. However, the current evidence base remains heterogeneous, ranging from exploratory biomarker studies and proof-of-concept experiments to a limited number of early-phase clinical trials. Consequently, promising findings obtained from in vitro systems or animal models should not be interpreted as direct evidence of clinical efficacy. Future studies should more clearly distinguish discovery, analytical validation, clinical validation, and therapeutic evaluation so that the translational maturity of individual exosome-based applications can be assessed more accurately.

A major priority is the establishment of reproducible and clinically applicable development frameworks. Variability in donor-cell source, culture conditions, isolation and purification procedures, cargo-loading strategies, and storage conditions can substantially influence vesicle identity, composition, potency, and batch-to-batch consistency. Therefore, exosome-based products will require well-defined critical quality attributes, standardized release criteria, and potency assays that are mechanistically linked to their intended diagnostic or therapeutic functions. In parallel, greater attention should be directed toward biodistribution, pharmacokinetics, dose–response relationships, long-term safety, immunogenicity, and potential off-target effects. For diagnostic applications, prospective validation in independent and multicenter cohorts will be essential to determine whether candidate biomarkers provide clinically meaningful advantages beyond existing tests. For therapeutic applications, rigorously designed clinical studies should establish optimal dosing, administration routes, therapeutic windows, and patient-selection strategies. Emerging technologies may help overcome several of these barriers. Microfluidic processing and automated analytical systems may improve reproducibility and reduce operator-dependent variability, whereas advanced bioengineering strategies may enable more consistent control over vesicle composition, cargo loading, and tissue targeting. Nevertheless, technological sophistication alone will not ensure successful translation. Progress will depend on coordinated efforts among basic scientists, clinicians, bioengineers, manufacturers, and regulatory authorities to harmonize terminology, analytical standards, manufacturing practices, and clinical evaluation criteria. With rigorous validation and transparent reporting, exosome-based diagnostics and therapeutics may progress from promising experimental concepts toward reliable and clinically meaningful components of precision medicine.

## Figures and Tables

**Table 1:** Common non-extracellular vesicle contaminants in exosome preparations and strategies for their detection and mitigation.

Contaminant	Potential Interference	Representative Detection Markers	Mitigation Strategies
Lipoproteins	Alter lipid signaling or overestimation of particle numbers	APOA1, APOB, APOE	SEC, DGC
Protein aggregates	Trigger immune responses	Total protein profile, aggregate-sensitive analysis	SEC, DGC, filtration
Soluble proteins	Produce EV-independent biological activity	Immunoglobulins, cytokines, Albumin	SEC, concentration controls
Cellular debris	Deliver intracellular proteins or nucleic acids	Calnexin, GM130	Differential centrifugation, filtration

Abbreviations: SEC, size exclusion chromatography; DGC, density gradient centrifugation.

**Table 2:** Representative clinical trials and pivotal preclinical studies of exosome-based therapeutic platforms.

Author/Year	Exosome Source	Cargo/Engineering Strategy	Disease Model	Key Outcome
**Clinical trials**				
Escudier et al., 2005 (Phase I) [50]	Autologous DEXs	MAGE-3 peptide loading	Advanced melanoma	Feasible and well tolerated; limited clinical response with immune activation in some patients.
Morse et al., 2005 (Phase I) [51]	Autologous DEXs	MAGE peptide loading	Advanced NSCLC	Well tolerated; immune responses were observed, but objective responses were limited.
Besse et al., 2016 (Phase II) [52]	IFN-γ-matured DEXs	MHC-associated tumor antigens	Advanced NSCLC	Primary endpoint not met; some disease stabilization and increased NK-cell activity were observed.
**Pivotal preclinical studies**				
Alvarez-Erviti et al., 2011 [22]	Dendritic cell exosomes	RVG–Lamp2b and BACE1 siRNA	Mouse brain delivery	Brain-targeted siRNA delivery reduced BACE1 expression.
Ohno et al., 2013 [53]	HEK293 exosomes	GE11 targeting and let-7a	EGFR-positive breast cancer	Enhanced tumor targeting and suppressed tumor growth.
Yang et al., 2017 [54]	Bone marrow MSC exosomes	RVG–Lamp2b and miR-124	Mouse cerebral ischemia	Promoted neurogenesis and reduced ischemic injury.
Song et al., 2017 [55]	IL-1β-primed umbilical cord MSC exosomes	miR-146a enrichment	Mouse sepsis	Promoted M2 polarization, reduced inflammation, and improved survival.
Cheng et al., 2018 [56]	HEK293 exosomes	Anti-CD3/anti-EGFR SMART-Exos	EGFR-positive breast cancer	Redirected T cells and enhanced antitumor activity.
Ji and Wang, 2024 [57]	Rat MSC exosomes	miR-21-5p delivery	Rat myocardial infarction	Reduced inflammation and apoptosis and preserved cardiac function.
Lin et al., 2024 [9]	hTERT adipose-derived MSC exosomes	PD-L1/HGF decoration with CXCR4 homing	Mouse ischemic stroke	Improved brain targeting, reduced injury, and enhanced neurological recovery.
Wiklander et al., 2024 [58]	HEK293T-derived EVs	Anti-HER2/anti-PD-L1 antibodies and doxorubicin	Breast cancer and melanoma	Improved tumor targeting and antitumor efficacy.

Abbreviations: DEXs, dendritic cell-derived exosomes; MSC, mesenchymal stromal/stem cell; RVG, rabies virus glycoprotein; NSCLC, non-small-cell lung cancer; MHC, major histocompatibility complex; NK, natural killer; EGFR, epidermal growth factor receptor; HGF, hepatocyte growth factor; PD-L1, programmed death-ligand 1.

**Table 3:** Comparison of major cargo-loading strategies for exosome engineering.

Loading Strategy	Representative Methods	Main Advantages	Major Limitations
Pre-loading	Genetic modification or incubation of donor cells with therapeutic agents	Preserves exosomal membrane integrity and enables endogenous cargo sorting	Variable loading efficiency; dependent on donor-cell uptake and sorting mechanisms
Passive post-loading	Incubation of isolated exosomes with hydrophobic or membrane- permeable compounds	Simple procedure with minimal membrane disruption	Generally low loading efficiency and limited cargo compatibility
Active post-loading	Electroporation, freeze–thaw cycles, chemical permeabilization, extrusion, sonication, and microfluidic loading	Improves cargo incorporation and supports diverse therapeutic payloads	May cause cargo aggregation, membrane damage, or alterations in vesicle function

Pre-loading introduces therapeutic cargo during exosome biogenesis, whereas post-loading incorporates cargo into previously isolated exosomes through passive or active approaches.

## Data Availability

No new data were created or analyzed in this study. Data sharing is not applicable to this article.
